# Raloxifene and progesterone neuroprotection in a female mouse model of Parkinson’s disease

**DOI:** 10.3389/fnins.2026.1870069

**Published:** 2026-09-09

**Authors:** Morgane M. André, Marc Morissette, Mélanie Bourque, Marie Rieux, Denis Soulet, Thérèse Di Paolo

**Affiliations:** 1 Centre de Recherche du CHU de Québec, Axe Neurosciences, Québec, QC, Canada; 2 Faculté de Pharmacie, Université Laval, Québec, QC, Canada

**Keywords:** female, MPTP, Parkinson’s disease, progesterone, raloxifene

## Abstract

**Introduction:**

Parkinson’s disease (PD) main pathological feature involves degeneration of dopamine (DA) neurons in the brain substantia nigra (SN). Female gonadal steroids are shown to be neuroprotective in animal models of PD and tested mainly in male and less in female animals. Since most women with PD are in menopause the present study tested ovariectomized (OVX) female mice as a model of the hormonal condition of menopause. This study sought the neuroprotective effect of the selective estrogen receptor modulator (SERM) raloxifene and progesterone alone and in combination to protect DA neurons in 1-methyl-4-phenyl-1,2,3,6- tetrahydropyridine (MPTP)-lesioned mice as a model of PD.

**Methods:**

Mice were treated with vehicle, raloxifene (2.5 mg/kg, b.i.d., subcutaneous), progesterone (1 μg, b.i.d., subcutaneous) and their combination for 10 days and administered MPTP (5.5 mg/kg, intraperitoneal) or saline on the 5th day; brains and uteri were collected thereafter.

**Results:**

Raloxifene treatment led to only small increases in uterine weights less than an average of intact mice uterine weights and no effect of progesterone. OVX MPTP-lesioned female mice showed a loss of striatal DA and metabolites content similarly prevented by treatments with raloxifene, progesterone and their combination. By contrast, striatal serotonin and metabolite remained unchanged. Striatal glial fibrillary acidic protein (GFAP), an astrogliosis marker, levels were elevated in MPTP-lesioned mice and this was similarly prevented with the hormonal treatment alone or in combination.

**Conclusion:**

Raloxifene and progesterone treatment was neuroprotective without excessive uterine stimulation in OVX mice supporting repurposing of these drugs for PD and their high translational value.

## 1 Introduction

The main pathological feature of Parkinson’s disease (PD) is the selective and progressive degeneration of dopamine (DA) neurons in the substantia nigra *pars compacta* (SNc) resulting in decreased dopaminergic input to the striatum the main recipient of afferents to the basal ganglia (Kalia and Lang, 2015). Approximately 5–10% of PD cases are attributable to genetic factors (Kalia and Lang, 2015; Jia et al., 2022) whereas the etiology of idiopathic PD is still unknown, but a variety of environmental and epigenetic factors are suspected to be involved (Kalia and Lang, 2015; Jia et al., 2022). There is no cure for PD but symptomatic treatments are available mainly L-3,4-dihydroxyphenylalanine (L-Dopa), the DA precursor, still remaining the gold standard treatment (Olanow and Stocchi, 2018). However, in the long-term L-Dopa treatment is associated with motor complications such as motor fluctuations and involuntary movements called L-Dopa-induced dyskinesia difficult to manage and reducing the quality of life of patients (Aradi and Hauser, 2020). Hence, given the increase of PD expected due to the aging population and the lack of treatment to cure PD, there is an urgent need to find new treatments acting at early stages of the disease to prevent or slow down its progression (Dorsey et al., 2018; Ou et al., 2021).

Epidemiological studies report sex differences in the prevalence, incidence, age of onset and severity of PD (Abraham et al., 2019). The prevalence of PD is ~2-fold higher in men and the onset of the disease in women is about 2 years later than in men (Cerri et al., 2019). The lower susceptibility of women to develop PD could be explained by exposure to female sex hormones, estrogen and progesterone (Frentzel et al., 2017; Bourque et al., 2019). Accordingly, at menopause where estrogen and progesterone levels are very low, deterioration of parkinsonian symptoms is observed while premenopausal women who underwent bilateral oophorectomy, especially at an early age, had an increased risk of parkinsonism and PD compared to women who did not undergo this surgery (Rocca et al., 2008, 2022; Pesce et al., 2022). Moreover, several clinical studies have shown that the risk of developing PD is reduced in women receiving hormone replacement therapy early after menopause (Bourque et al., 2019). Numerous studies in different animal models of PD report the beneficial influence of estrogens and more particularly estradiol, to protect DA neurons against various neurotoxins (Litim et al., 2016; Bourque et al., 2024). Nevertheless, there is a significant risk of long-term treatment with estrogens as such as an increased risk of breast cancer, venous thromboembolism, coronary artery disease and in men with a feminizing effect and an increased risk to develop cancer (Kulkarni et al., 2013; Flores et al., 2021). Selective estrogen receptor modulators (SERMs), such as raloxifene, could circumvent these problems (Veenman, 2020; Farkas et al., 2022).

Raloxifene a second-generation SERM is prescribed to treat and prevent osteoporosis and is also approved to reduce the risk of invasive breast cancer in postmenopausal women (Veenman, 2020). Raloxifene acts as an estrogen antagonist on estrogen receptors (ER) in breast and reproductive tissues while it shows estrogenic agonist effects on bone, lipid metabolism, and brain (Gizzo et al., 2013; Lee et al., 2013). An important feature of long-term treatment with raloxifene is the beneficial effects observed in the brain of postmenopausal women and elderly men with no evident adverse hormonal side effects and no feminizing effects in men (Veenman, 2020). Interestingly, raloxifene shows neuroprotective effects on nigrostriatal DA neurons in animal models of PD such as MPTP-lesioned male mice or ovariectomized female rats lesioned with 6-OHDA (Grandbois et al., 2000; Callier et al., 2001; Baraka et al., 2011; Bourque et al., 2014; Baez-Jurado et al., 2019). Moreover, peripheral neuroprotective and immunomodulatory effects of raloxifene were also shown in myenteric DA neurons (Poirier et al., 2016). Therefore, raloxifene appears to be an excellent therapeutic alternative to estrogen for the prevention and/or reduction of PD progression in both men and women.

Neuroprotective effects of estrogenic compounds, mainly 17β-estradiol, have been widely reported in animal models of PD (Callier et al., 2000; Morissette et al., 2008; Bourque et al., 2009) while the neuroprotective effects of progesterone have been less studied (Bourque et al., 2024). However, beneficial effects of progesterone were demonstrated in animal models of several neurological disorders such as brain injury, ischemia, spinal cord and peripheral nerve injury, demyelinating diseases, neuromuscular disorders, and epilepsy (Deutsch et al., 2013). Several clinical reports also highlighted the beneficial effects of progesterone in patients with traumatic brain injury (Wright et al., 2007; Xiao et al., 2008). A neuroprotective effect of progesterone is documented on DA neurons in MPTP-lesioned male mice (Grandbois et al., 2000; Callier et al., 2001; Bourque et al., 2016). More recently, progesterone has shown peripheral neuroprotective effects in DA neurons in the myenteric plexus (Jarras et al., 2020). Interestingly, treatment with 17β-estradiol, progesterone and their combination in MPTP-lesioned male mice were reported to similarly prevent striatal DA loss mice (Morissette et al., 2008). To our knowledge the combined treatment with estradiol and progesterone has not been reported in a female rodent model of PD intact or OVX. Moreover, the combination of a SERM with progesterone has not been reported in animal models of PD.

PD typically develops around the age of 60 (Kalia and Lang, 2015) when most women are postmenopausal and have low circulating estradiol levels (Santoro et al., 2021). Therefore, the present study was performed in ovariectomized female mice to model ovarian steroid loss as in menopause. The goal of the present study was to investigate nigrostriatal DA neurons neuroprotection by raloxifene and progesterone treatments in ovariectomized MPTP-lesioned female mice. Based on the male/female difference in PD prevalence the rationale is that a combination of the major female sex steroids 17β-estradiol and progesterone is neuroprotective. Because of a risk of cancer with 17β-estradiol and it is feminizing for men the non-feminizing estrogenic drug raloxifene is proposed in combination with progesterone. Since neuroinflammation is proposed in the pathophysiology of PD and that it is documented that estrogens and progesterone have anti-inflammatory activity, we explored the implication of neuroinflammation (specifically here astrogliosis labelled with GFAP) in the DA neuroprotection observed with raloxifene, progesterone and raloxifene +progesterone treatments. The combination was intended to determine if there is an additive/synergistic neuroprotective effect or whether combined treatment retains the neuroprotective effects observed with the individual agents or the combination opposes the individual agents.

## 2 Materials and methods

### 2.1 Animals

Twelve-week-old ovariectomized female C57BL/6 N mice were purchased from Charles River Canada (Montreal, QC, Canada) and upon arrival they were housed in cages under controlled conditions at 22 °C with a 12 h light/12 h dark cycle with food and water *ad libitum*. The mice had an acclimatization period of 1 week before drug or vehicle administration and the experiment was started 6 weeks after their ovariectomy. These mice were divided into 8 experimental groups including: ovariectomized (OVX) female mice treated with saline + vehicle (*n* = 15, thereafter named vehicle), saline + raloxifene (*n* = 10, thereafter named raloxifene), saline + progesterone (*n* = 10, thereafter named progesterone), saline + raloxifene + progesterone (*n* = 10, thereafter named raloxifene + progesterone), MPTP + vehicle (*n* = 13, thereafter named MPTP), MPTP + raloxifene (*n* = 10), MPTP + progesterone (*n* = 10), or MPTP + raloxifene + progesterone (*n* = 10). The number of mice per group was based on our previous experiment in the brain of intact male mice that received MPTP and raloxifene treatment (Bourque et al., 2014). The Université Laval Animal Care Committee approved these animal studies (protocol 2024–1509). All efforts were made to minimize animal suffering and to reduce the number of mice used.

### 2.2 Drug treatment

Mice administered with raloxifene (Cat. No. 2280, Tocris Bioscience, Bristol, UK; 2.5 mg/kg, subcutaneous) or progesterone (Cat. No. P0130, MilliporeSigma, Burlington, MA, USA; 1 μg/mouse, subcutaneous) or vehicle only (0.9% saline with 0.3% gelatin, subcutaneous) or their combination at the same doses were injected twice daily for 10 days. MPTP treatment (Cat. No. M269025MG, Fisher Scientific, Waltham, MA, USA) consisted of four intraperitoneal injections (5.5 mg/kg in 0.9% saline per injection, total dose of MPTP of 22 mg/kg per mouse) at 2-h intervals on Day 5. Unlesioned mice received four intraperitoneal injections of 0.9% saline at 2-h intervals on Day 5. The MPTP dose of the present study was chosen to produce a partial lesion of the DA nigrostriatal pathway (Grandbois et al., 2000; Callier et al., 2001; Litim et al., 2015) to model early stages of PD. Raloxifene and progesterone were investigated in this study at a dose that we previously showed to protect against MPTP toxicity brain DA of intact male mice (Bourque et al., 2014; Bourque et al., 2016).

### 2.3 Brain tissue preparation

On day 11, mice were deeply anesthetized with ketamine-xylazine (90/10 mg/mL) (Fish et al., 2008) before cardiac puncture and transcardial perfusion with 0.1 M PBS (137 mM NaCl, 2.7 mM KCl, 4.3 mM Na_2_HPO_4_, 1.47 mM KH_2_PO_4_, pH 7.4). The mice uteri were immediately removed after euthanasia, freed from connective and adipose tissue, and weighed. The brain was quickly removed and separated in two hemispheres. The first hemisphere was rapidly placed in isopentane on dry ice (− 40 °C) and the anterior part of the striatum was dissected to assay biogenic amine and their metabolites contents. The second hemisphere was post-fixed with 4% paraformaldehyde for 48 h, placed in 0.1 M PBS containing 20% (w/v) sucrose and stored at 4 °C. Fixed brains were frozen with dry ice/ethanol mixture, mounted on a microtome (Leica Microsystems Inc., ON, Canada) and cut into 30 μm thick coronal sections. The collected fixed brain sections were immersed in tissue cryoprotectant solution (0.05 M PBS pH 7.3, 30% ethylene glycol and 20% glycerol) and stored at - 20 °C until use for immunofluorescence.

### 2.4 Striatal biogenic amine assay

The left anterior striata were dissected, homogenized in 250 μL of 0.1 N HClO_4_ at 4 °C and then centrifuged at 10000.*g* for 20 min (4 °C) to precipitate proteins. The striatal contents of DA and its metabolites 3,4- dihydroxyphenylacetic acid (DOPAC), 3-methoxytyramine (3-MT) and homovanillic acid (HVA) as well as serotonin (5-HT) and its metabolite 5-hydroxyindoleacetic acid (5-HIAA) were measured by HPLC with electrochemical detection (examples of chromatograms included in Supplementary Figure 1). Supernatants of striatal tissue were directly injected into the chromatograph under conditions we previously reported (Isenbrandt et al., 2021).

### 2.5 GFAP immunofluorescence

GFAP positive astrocytes were measured using an immunofluorescence-based approach. Free-floating 30 μm thick brain sections containing the striatum (4–7 brain sections) were first incubated overnight at room temperature with primary antibody mouse anti-GFAP (Millipore; catalogue no. MAB360; dilution 1:500). The next day, brain sections were stained with secondary antibody donkey anti-mouse Alexa Fluor 488 (Life Technologies; catalogue no. A21202; dilution 1:1000). DAPI nuclear counterstaining (Invitrogen Corporation) was performed to detect nuclei. Slide scanning was performed with an Axio Scan.Z1 Digital Slide Scanner (Zeiss Canada) and images were acquired with a 5X objective lens (NA 0.25). Contour of the region was drawn, and GFAP intensities were assessed by measuring the mean pixel intensity in the delimited areas using ImageJ (Fiji) software, version 2.16.0/1.54p.

High-magnification photomicrographs of astrogliosis in the caudate-putamen region (identified by the presence of striosomes) were acquired using a confocal laser-scanning microscope (Olympus Fluoview IX81 FV1000, Olympus Canada, Richmond Hill, ON, Canada) equipped with a 60×/1.4 NA oil-immersion objective (1.1 × optical zoom) and a 488-nm laser line. Image stacks consisted of eight z-planes acquired at 0.41-μm intervals and were processed using maximum-intensity projection. All acquisition parameters were kept constant across samples.

### 2.6 Statistical analyses

The statistical analyses used GraphPad Prism software (v.6.0b) for Apple Macintosh Computer. Results were analyzed using a one-way and a two-way ANOVA followed by a Sidak’s (one-way ANOVA) or Tukey’s (two-way ANOVA) *post-hoc* test. The two factors are treatment (raloxifene, progesterone, raloxifene + progesterone) and lesion (non-lesioned saline-treated mice or MPTP-lesioned mice). When the two-way ANOVA gave no interaction between the two variables, a one-way ANOVA was presented. The relationship between the GFAP striatal levels and DA contents was evaluated with a *Pearson’s correlation coefficient*. A *p* ≤ 0.05 was required for the results to be considered statistically significant.

## 3 Results

### 3.1 Uterine weights

**Figure 1:**
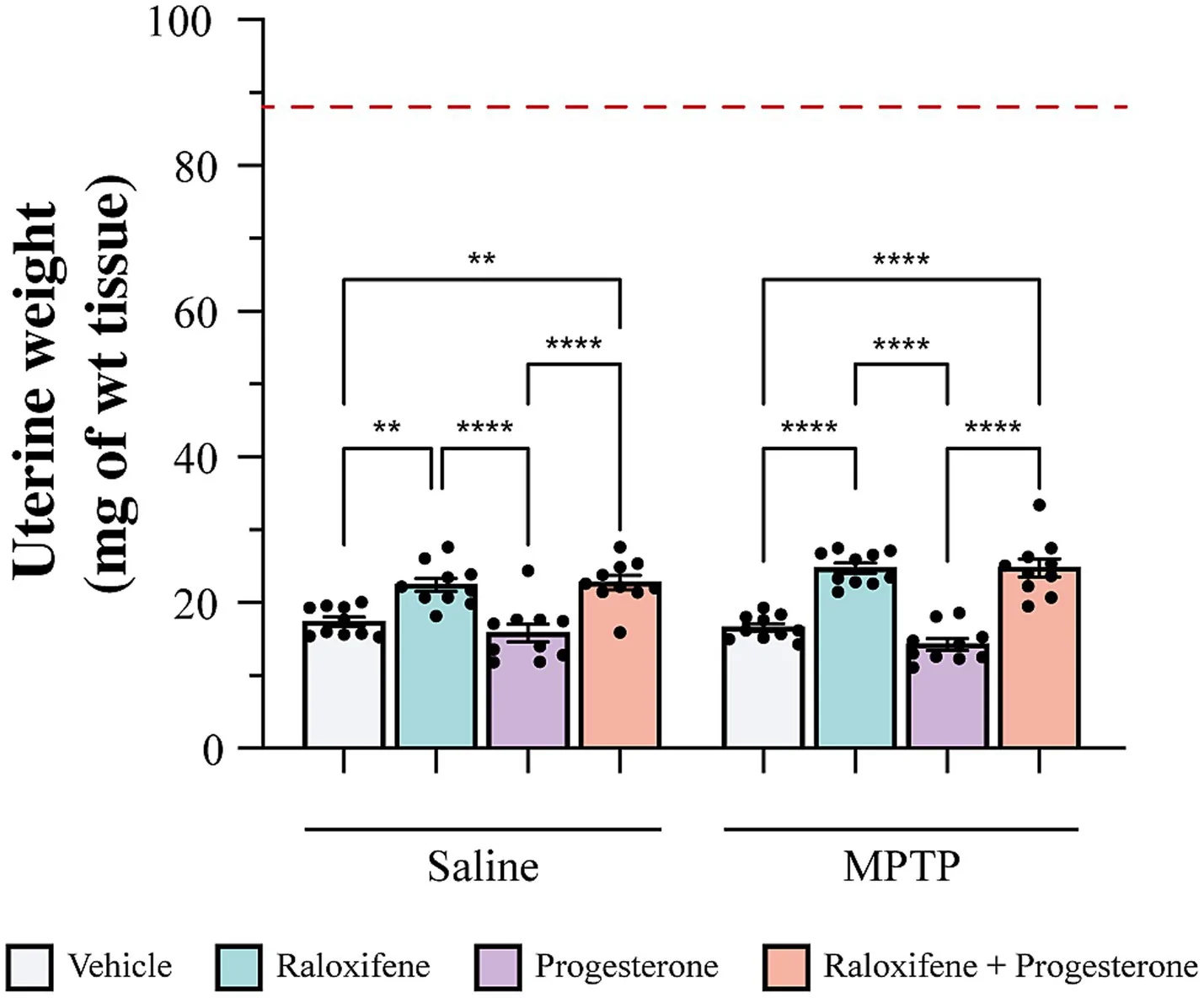
Effects of a chronic treatment with raloxifene and progesterone on uterine weight of unlesioned saline-treated and MPTP-lesioned ovariectomized female mice. Values shown are the means (mg of wet tissue) ± standard error of the mean (SEM) of 10–15 mice per group and points show data for individual mouse. ** *p* < 0.01 and **** *p* < 0.0001. A horizontal red dashed line at the top marks intact mouse weight for reference.

Uterine weights were in general lower than the average of intact mouse values as expected because of ovariectomy-induced low estrogen levels and showed effects of treatments (*F* (7, 72) = 22.05, *p* < 0.0001) (Figure 1). In saline-treated and MPTP-lesioned mice raloxifene alone or with progesterone induced a small but significant increase of uterine weights (vehicle vs. raloxifene and vehicle vs. raloxifene+progesterone respectively, saline-treated mice: *p* = 0.0048; *p* = 0.0023 and MPTP-lesioned mice: *p* < 0.0001; *p* < 0.0001) whereas progesterone left these weights unchanged (saline-treated mice: *p* = 0.9227 and MPTP-lesioned mice: *p* = 0.6130).

### 3.2 Striatal biogenic amines content

**Figure 2:**
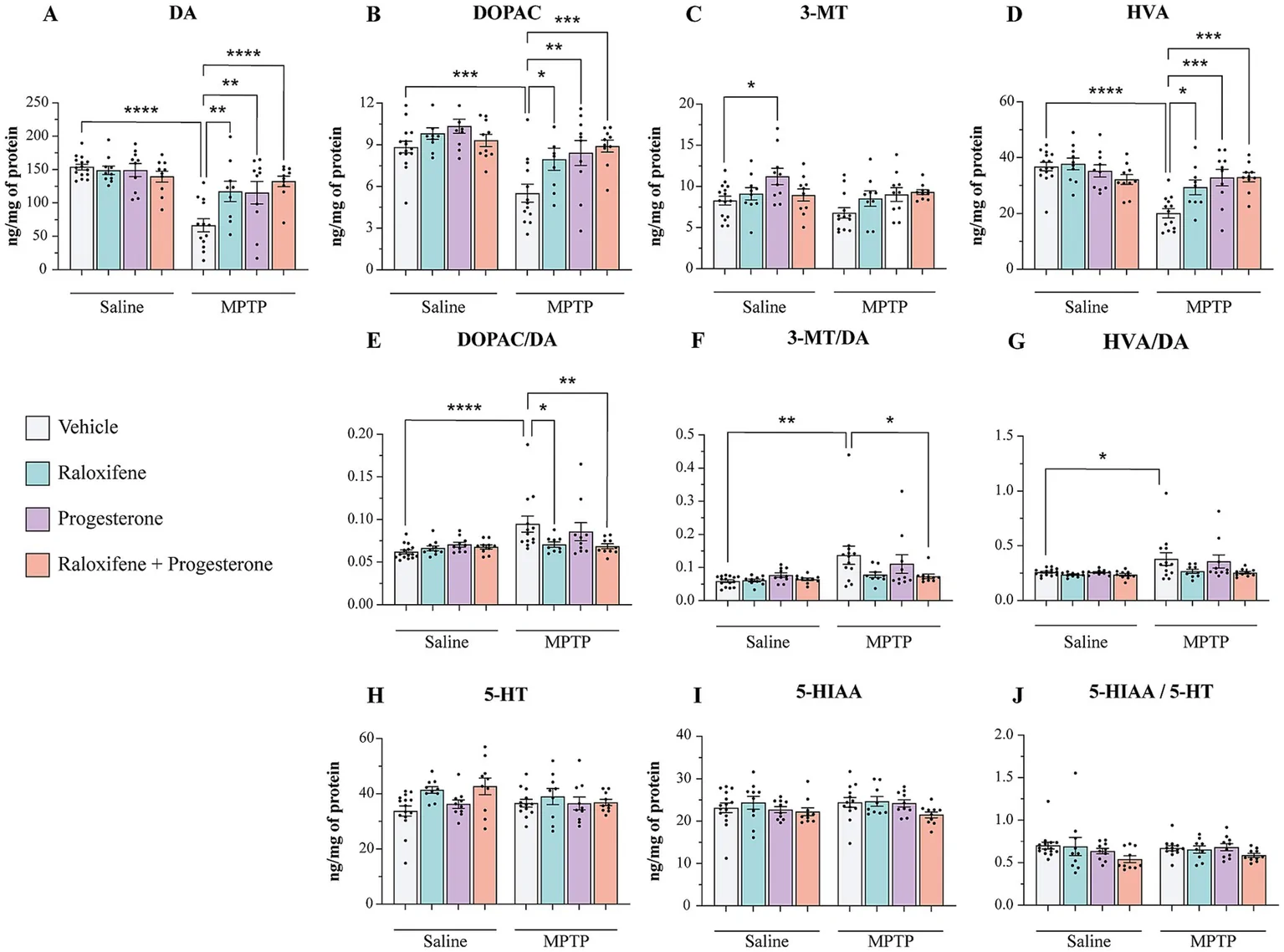
Effects of a chronic treatment with raloxifene and progesterone on striatal **(A)** dopamine (DA), and its metabolites **(B)** 3,4-dihydroxyphenylacetic acid (DOPAC), **(C)** 3-methoxytyramine (3-MT), **(D)** homovanillic acid (HVA) contents in addition to their ratios **(E)** DOPAC/DA, **(F)** 3-MT/DA, **(G)** HVA/DA as well as **(H)** serotonin (5-HT), **(I)** its metabolite 5-hydroxyindoleacetic acid (5-HIAA) contents and **(J)** 5-HIAA/5-HT of unlesioned saline-treated and MPTP-lesioned ovariectomized female mice. Values shown are the means (ng/mg of proteins) ± SEM of 10–15 mice per group and points show data for individual mouse. *p < 0.05, **p < 0.01, ***p < 0.001 and ****p < 0.0001.

A significant effect of the MPTP lesion and the hormonal treatments was observed for DA and its metabolites (DA: two-way ANOVA: treatment: *F* (3, 79) = 3.339, *p* = 0.0235; lesion: *F* (1, 79) = 31.64, *p* < 0.0001; interaction: *F* (3, 79) = 6.513, *p* = 0.0005; DOPAC: *F* (7, 79) = 6.879, *p* < 0.0001; 3-MT: *F* (7, 79) = 3.060, *p* = 0.0067; HVA: treatment: *F* (3, 79) = 4.259*, p* = 0.0077; lesion: *F* (1, 79) = 21.05, *p* < 0.0001; interaction: *F* (3, 79) = 7.283, *p* = 0.0002) (Figures 2A–D). Treatments with raloxifene, progesterone and their combination overall left unchanged striatal DA and its metabolites contents of unlesioned saline treated mice except for an increase of the intermediate metabolite 3-MT with the progesterone treatment. The MPTP lesion induced a decrease of striatal DA, DOPAC and HVA while this was not significant for 3-MT. Treatments with raloxifene, progesterone and their combination prevented to a similar extent the striatal DA and metabolites MPTP-induced losses showing no synergy/additive nor opposing effects. The effects of lesion and treatments in the striatum of DA was compared to 5-HT and its metabolites striatal contents (5-HT: *F* (7, 79) = 2.308, *p* = 0.0341; 5-HIAA: *F* (7, 79) = 1.115, *p* = 0.362) (Figures 2H,I). The effect of the MPTP lesion was specific to DA, striatal 5-HT remaining unchanged in untreated and hormonal MPTP-lesioned mice. Raloxifene increased 5-HT contents of unlesioned mice while progesterone had no effect. Striatal 5-HIAA remained unchanged by the lesion and the treatments.

The ratios DOPAC/DA (two-way ANOVA: treatment: *F* (3, 79) = 1.951, *p* = 0.1282; lesion: *F* (1, 79) = 10.37, *p* = 0.0019; interaction: *F* (3, 79) = 3.444, *p* = 0.0206), 3-MT/DA (*F* (7, 79) = 3.482, *p* = 0.0026), HVA/DA (*F* (7, 79) = 3.054, *p* = 0.0067), estimating the turnover of DA are shown in Figures 2E–G. The ratio 5-HIAA/5-HT (*F* (7, 79) = 1.27, *p* = 0.2758) displaying the turnover of 5-HT is shown in Figure 2J. The treatments left unchanged the DA turnover in unlesioned mice. The MPTP lesion increased DA turnover that was similarly prevented with the treatments. The turnover of 5-HT was unchanged by the lesion and treatments.

### 3.3 Striatal GFAP content

**Figure 3:**
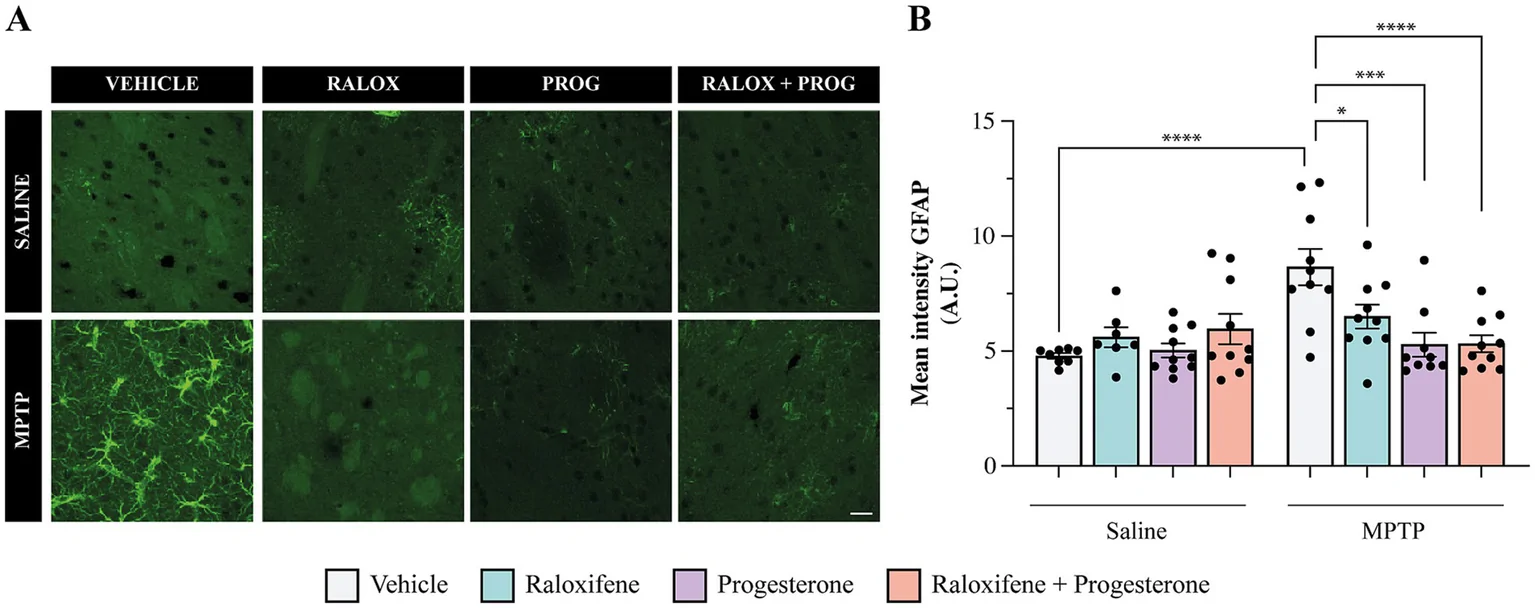
**(A)** Representative high-resolution photomicrographs of GFAP-positive astrocytes in the striatum, illustrating the effects of chronic treatment with raloxifene or progesterone in unlesioned saline-treated and MPTP-lesioned ovariectomized female mice (confocal microscopy). Scale bar = 20 μm. **(B)** Histograms showing the mean pixel intensity ± SEM for 8–10 mice per group and points show data for individual mouse. Quantification was performed on large-field-of-view images acquired using whole-slide scanning microscopy. A.U., arbitrary unit; PROG, progesterone; RALOX, raloxifene. *p < 0.05, ***p < 0.001, and ****p < 0.0001.

Examples of GFAP, an astrocyte marker, labelling by immunofluorescence in the striatum is shown in Figure 3A. Histograms of GFAP results shown in Figure 3B report significant effect of the lesion and treatments (two-way ANOVA: treatment: *F* (3, 66) = 3.316, *p* = 0.0303; lesion: *F* (1, 66) = 8.522, *p* = 0.0048; interaction: *F* (3, 66) = 6.879, *p* = 0.0004). Treatments with raloxifene, progesterone and their combination overall left unchanged striatal GFAP levels of unlesioned saline treated mice (respectively *p* = 0.7620, *p* = 0.9890 and *p* = 0.4150 compared to controls). The MPTP lesion induced an increase of striatal GFAP levels that was similarly prevented with the hormonal treatments (compared to MPTP, raloxifene: *p* = 0.0187, progesterone: *p* = 0.0001 and raloxifene+progesterone: *p* < 0.0001).

### 3.4 Striatal correlation of GFAP with DA

**Figure 4:**
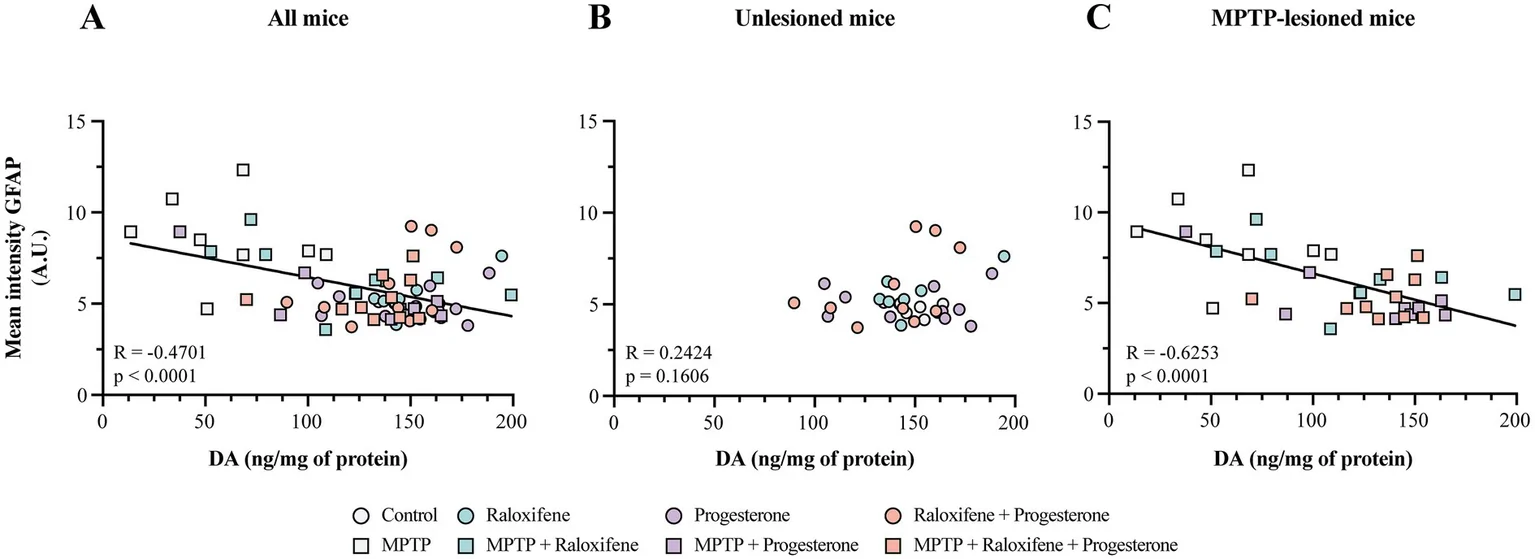
Correlations between striatal DA contents with striatal GFAP levels in **(A)** all unlesioned saline-treated and MPTP-lesioned ovariectomized female mice treated with raloxifene and progesterone, **(B)** correlation including only unlesioned saline-treated mice, and **(C)** correlation including all the groups of MPTP-lesioned mice. Each symbol in the correlations represents values for an individual mouse.

A significant negative correlation was observed between individual mouse values of striatal GFAP levels and DA contents of all these mice (*R* = −0.4701, P (two-tailed) < 0.0001, *n* = 71) (Figure 4A). When only the unlesioned mice were compared, no significant correlation was obtained (*R* = 0.2424, P (two-tailed) = 0.1606, *n* = 35) (Figure 4B) and when all the MPTP groups were compared a significant negative correlation was obtained (*R* = −0.6619, P (two-tailed) < 0.0001, *n* = 44) (Figure 4C).

## 4 Discussion

The present study showed similar neuroprotection of striatal DA in MPTP-lesioned mice by raloxifene, progesterone and their combination associated with prevention of astrogliosis in OVX female mice showing no synergy/additive nor opposing effects of the combination. Menopause with low gonadal steroid levels is the hormonal status of most women with idiopathic PD and was modeled with ovariectomy in mice. OVX female mice have reduced uterine weights compared to intact female mice and the brain neuroprotection with raloxifene, progesterone and their combination increased uterine weights but to values much less than in intact mice.

Raloxifene, progesterone and their combination each protected maximally striatal DA and its metabolites, and this was specific for this neurotransmitter, 5-HT and its metabolite remaining unchanged. This was also associated with a maximal prevention of the MPTP-induced increase of striatal GFAP.

Behavior measures for these mice were not performed since under the conditions of moderate DA loss as in the present study we previously reported no motor impairment using the latency to fall in the rotarod test as well as the mean speed and distance traveled in the open-field test of MPTP-lesioned mice (Litim et al., 2017a). We aim to investigate an early pre-symptomatic stage of PD when hormonal treatment is effective. Willard et al., 2015 investigated the differential degradation of motor deficits during gradual DA depletion with 6-hydroxydopamine in mice and showed spontaneous locomotion, measured in an open field, was robust to loss of DA until ~70% of striatal DA was lost. Beyond this point, additional DA loss caused a sharp decline in motor performance. This in accordance with our results (Litim et al., 2017a) and supports the present results with a 57% DA depletion to be in a pre-symptomatic stage. Animal models of PD are in accordance with human data reporting that motor symptoms such as tremors, rigidity, bradykinesia, freezing, and balance instability typically do not become overt enough to diagnose PD until dopaminergic loss exceeds 70% in the striatum (Bernheimer et al., 1973; Riederer and Wuketich, 1976; Deumens et al., 2002; Fahn, 2003).

At the doses of raloxifene and progesterone investigated we did not measure an additive or synergistic effect of these drugs, a maximal effect being observed. A lower dose of each drug would be needed to test synergy.

A raloxifene + progesterone treatment has not been reported in a rodent model of PD but the combination of 17β-estradiol + progesterone treatment in MPTP-lesioned male mice was shown to similarly protect striatal DA than the individual steroid treatment (Morissette et al., 2008). A common mechanism of action to reduce inflammation likely explains the neuroprotective effects of raloxifene (or 17β-estradiol) and progesterone. It is well documented that estrogens have anti-inflammatory activity (Lu et al., 2025), notably in PD models (Cote et al., 2015; Poirier et al., 2022) and that estrogenic activity of raloxifene is also associated with anti-inflammatory activity both *in vivo* and *in vitro* (Poirier et al., 2022). Progesterone is also reported to have anti-inflammatory activity (Litim et al., 2017b; Fedotcheva and Shimanovsky, 2025). However, the molecular mechanisms of the neuroprotective and anti-inflammatory actions of progesterone remain poorly understood, it is not clear whether progesterone metabolites could also be involved indirectly (Fedotcheva and Shimanovsky, 2025).

A limitation of the present study is the use of young adult female mice that does not take into consideration aging and its effect in the brain, whereas most patients with PD are older. We used ovariectomy to reduce blood levels of ovarian steroids to model menopause. However, ovariectomy was reported to change neuroactive steroid levels in the rat nervous system and not necessarily reflected by changes in plasma levels (Caruso et al., 2010). Moreover, long-term ovariectomy in rats induced changes in brain neuroactive steroids levels that, in some cases, are different to those induced by short-term ovariectomy (Caruso et al., 2010). OVX rodents allow to assess the effect of abrupt and extensive peripheral ovarian hormone loss and does not model the gradual, transitional nature of the menopausal process in women. The progressive transition to a follicle-deplete, ovary-intact state, as achieved with the VCD (4-vinylcyclohexene diepoxide) model more closely approximates the physiological human progression through peri- and post-menopause than the abrupt hormone loss induced by ovariectomy (Koebele and Bimonte-Nelson, 2016). The aromatase inhibitor letrozole was also used instead of ovariectomy to investigate reduced estradiol levels in neuroprotection (Zarate et al., 2025). Nevertheless, as we reported previously (Isenbrandt et al., 2021) ovariectomy affects the brain DA response to MPTP supporting a neuroprotective effect of ovarian steroids. We previously showed that the same dose of MPTP in sham-operated female mice led to significantly less striatal DA loss (reduced to 84% of control values) compared to about half in OVX female mice (reduced to 51% of control values) (Isenbrandt et al., 2021).

Raloxifene or progesterone is used safely in humans and offer the possibility to be repositioned for PD treatment in women. Since raloxifene is non-feminizing and was tested in men (de Boer et al., 2018) it could also be useful for men with PD as well as progesterone. Raloxifene available as a generic drug, is used to treat breast cancer, osteoporosis, vertebral compression fractures and postmenopausal symptoms (An, 2016). Raloxifene does not increase risk of cancer in reproductive organs. Motor impairment in PD patients often leads to more falls and raloxifene brings an added benefit for bones. Meta-analyses on the use of raloxifene as adjunctive treatment for postmenopausal women with schizophrenia (Wang et al., 2018; Zhu et al., 2018) or in men and women with schizophrenia (de Boer et al., 2018) show that it is effective and safe in improving psychotic symptoms. Raloxifene has been widely used in humans for many years with limited side effects (venous thromboembolism) (0.24–1.1%) and possible stroke (0.95–2.9%) (Ellis et al., 2015). Progesterone treatment is generally considered safe for humans, but it can cause side effects and has some counter-indications (Xu et al., 2026). Interaction between estradiol and progesterone have been studied in various contexts, and the results suggest that these hormones have both synergistic and antagonistic effects on different biological processes. For example, in the hippocampus, volume of CA1, a structure related to learning and memory processes, has been correlated positively with estradiol and negatively with progesterone (Zsido et al., 2023). Given conflicting data in women with history of breast cancer or those taking estrogen therapy, progesterone should be used with caution since it can interact with estradiol (Trabert et al., 2020). Nevertheless, considering that raloxifene does not increase risk of cancer in reproductive organs, the combined treatment of raloxifene + progesterone should be safe, but its clinical safety has yet to be directly tested. Thus, as a follow-up experiment, it could be valuable to test lower doses of raloxifene and progesterone to determine whether the combined treatment could have an enhanced neuroprotective and immunomodulatory effect.

## Data Availability

The raw data supporting the conclusions of this article will be made available by the authors, without undue reservation.

## References

[ref1] Abraham D. S. Gruber-Baldini A. L. Magder L. S. McArdle P. F. Tom S. E. Barr E. . (2019). Sex differences in Parkinson's disease presentation and progression. Parkinsonism Relat. Disord. 69, 48–54. doi: 10.1016/j.parkreldis.2019.10.019, 31677455 PMC6982644

[ref2] An K. C. (2016). Selective Estrogen Receptor Modulators. Asian Spine J. 10, 787–791. doi: 10.4184/asj.2016.10.4.787, 27559463 PMC4995266

[ref3] Aradi S. D. Hauser R. A. (2020). Medical management and prevention of motor complications in Parkinson's disease. Neurotherapeutics 17, 1339–1365. doi: 10.1007/s13311-020-00889-4, 32761324 PMC7851275

[ref4] Baez-Jurado E. Rincon-Benavides M. A. Hidalgo-Lanussa O. Guio-Vega G. Ashraf G. M. Sahebkar A. . (2019). Molecular mechanisms involved in the protective actions of selective estrogen receptor modulators in brain cells. Front. Neuroendocrinol. 52, 44–64. doi: 10.1016/j.yfrne.2018.09.001, 30223003

[ref5] Baraka A. M. Korish A. A. Soliman G. A. Kamal H. (2011). The possible role of estrogen and selective estrogen receptor modulators in a rat model of Parkinson's disease. Life Sci. 88, 879–885. doi: 10.1016/j.lfs.2011.03.010, 21420980

[ref6] Bernheimer H. Birkmayer W. Hornykiewicz O. Jellinger K. Seitelberger F. (1973). Brain dopamine and the syndromes of Parkinson and Huntington. Clinical, morphological and neurochemical correlations. J. Neurol. Sci. 20, 415–455. doi: 10.1016/0022-510x(73)90175-5, 4272516

[ref7] Bourque M. Dluzen D. E. Di Paolo T. (2009). Neuroprotective actions of sex steroids in Parkinson's disease. Front. Neuroendocrinol. 30, 142–157. doi: 10.1016/j.yfrne.2009.04.014, 19410597

[ref8] Bourque M. Morissette M. Al Sweidi S. Caruso D. Melcangi R. C. Di Paolo T. (2016). Neuroprotective effect of progesterone in MPTP-treated male mice. Neuroendocrinology 103, 300–314. doi: 10.1159/000438789, 26227546

[ref9] Bourque M. Morissette M. Di Paolo T. (2014). Raloxifene activates G protein-coupled estrogen receptor 1/Akt signaling to protect dopamine neurons in 1-methyl-4-phenyl-1,2,3,6-tetrahydropyridine mice. Neurobiol. Aging 35, 2347–2356. doi: 10.1016/j.neurobiolaging.2014.03.017, 24726471

[ref10] Bourque M. Morissette M. Di Paolo T. (2019). Repurposing sex steroids and related drugs as potential treatment for Parkinson's disease. Neuropharmacology 147, 37–54. doi: 10.1016/j.neuropharm.2018.04.005, 29649433

[ref11] Bourque M. Morissette M. Di Paolo T. (2024). Neuroactive steroids and Parkinson's disease: review of human and animal studies. Neurosci. Biobehav. Rev. 156:105479. doi: 10.1016/j.neubiorev.2023.105479, 38007170

[ref12] Callier S. Morissette M. Grandbois M. Di Paolo T. (2000). Stereospecific prevention by 17beta-estradiol of MPTP-induced dopamine depletion in mice. Synapse 37, 245–251. doi: 10.1002/1098-2396(20000915)37:4<245::AID-SYN1>3.0.CO;2-5, 10891861

[ref13] Callier S. Morissette M. Grandbois M. Pelaprat D. Di Paolo T. (2001). Neuroprotective properties of 17beta-estradiol, progesterone, and raloxifene in MPTP C57Bl/6 mice. Synapse 41, 131–138. doi: 10.1002/syn.1067, 11400179

[ref14] Caruso D. Pesaresi M. Maschi O. Giatti S. Garcia-Segura L. M. Melcangi R. C. (2010). Effect of short-and long-term gonadectomy on neuroactive steroid levels in the central and peripheral nervous system of male and female rats. J. Neuroendocrinol. 22, 1137–1147. doi: 10.1111/j.1365-2826.2010.02064.x, 20819120

[ref15] Cerri S. Mus L. Blandini F. (2019). Parkinson's disease in women and men: what's the difference? J. Parkinsons Dis. 9, 501–515. doi: 10.3233/JPD-191683, 31282427 PMC6700650

[ref16] Cote M. Bourque M. Poirier A. A. Aube B. Morissette M. Di Paolo T. . (2015). GPER1-mediated immunomodulation and neuroprotection in the myenteric plexus of a mouse model of Parkinson's disease. Neurobiol. Dis. 82, 99–113. doi: 10.1016/j.nbd.2015.05.017, 26051538

[ref17] de Boer J. Prikken M. Lei W. U. Begemann M. Sommer I. (2018). The effect of raloxifene augmentation in men and women with a schizophrenia spectrum disorder: a systematic review and meta-analysis. NPJ Schizophr. 4:1. doi: 10.1038/s41537-017-0043-3, 29321530 PMC5762671

[ref18] Deumens R. Blokland A. Prickaerts J. (2002). Modeling Parkinson's disease in rats: an evaluation of 6-OHDA lesions of the nigrostriatal pathway. Exp. Neurol. 175, 303–317. doi: 10.1006/exnr.2002.7891, 12061862

[ref19] Deutsch E. R. Espinoza T. R. Atif F. Woodall E. Kaylor J. Wright D. W. (2013). Progesterone's role in neuroprotection, a review of the evidence. Brain Res. 1530, 82–105. doi: 10.1016/j.brainres.2013.07.014, 23872219

[ref20] Dorsey E. R. Sherer T. Okun M. S. Bloem B. R. (2018). The emerging evidence of the Parkinson pandemic. J. Parkinsons Dis. 8, S3–S8. doi: 10.3233/JPD-181474, 30584159 PMC6311367

[ref21] Ellis A. J. Hendrick V. M. Williams R. Komm B. S. (2015). Selective estrogen receptor modulators in clinical practice: a safety overview. Expert Opin. Drug Saf. 14, 921–934. doi: 10.1517/14740338.2015.1014799, 25936229

[ref22] Fahn S. (2003). Description of Parkinson's disease as a clinical syndrome. Ann. N. Y. Acad. Sci. 991, 1–14. doi: 10.1111/j.1749-6632.2003.tb07458.x, 12846969

[ref23] Farkas S. Szabo A. Hegyi A. E. Torok B. Fazekas C. L. Ernszt D. . (2022). Estradiol and estrogen-like alternative therapies in use: the importance of the selective and non-classical actions. Biomedicine 10:861. doi: 10.3390/biomedicines10040861, 35453610 PMC9029610

[ref24] Fedotcheva T. A. Shimanovsky N. L. (2025). Neurosteroids progesterone and Dehydroepiandrosterone: molecular mechanisms of action in neuroprotection and Neuroinflammation. Pharmaceuticals (Basel) 18:945. doi: 10.3390/ph18070945, 40732235 PMC12298252

[ref25] Fish R. E. Brown M. J. Danneman P. J. Karas A. Z. (2008). Anesthesia and Analgesia in Laboratory Animals. 2nd Edn. Amsterdam: Academic Press.

[ref26] Flores V. A. Pal L. Manson J. E. (2021). Hormone therapy in menopause: concepts, controversies, and approach to treatment. Endocr. Rev. 42, 720–752. doi: 10.1210/endrev/bnab011, 33858012

[ref27] Frentzel D. Judanin G. Borozdina O. Klucken J. Winkler J. Schlachetzki J. C. M. (2017). Increase of reproductive life span delays age of onset of Parkinson's disease. Front. Neurol. 8:397. doi: 10.3389/fneur.2017.00397, 28871235 PMC5566617

[ref28] Gizzo S. Saccardi C. Patrelli T. S. Berretta R. Capobianco G. Di Gangi S. . (2013). Update on raloxifene: mechanism of action, clinical efficacy, adverse effects, and contraindications. Obstet. Gynecol. Surv. 68, 467–481. doi: 10.1097/OGX.0b013e31828baef9, 23942473

[ref29] Grandbois M. Morissette M. Callier S. Di Paolo T. (2000). Ovarian steroids and raloxifene prevent MPTP-induced dopamine depletion in mice. Neuroreport 11, 343–346. doi: 10.1097/00001756-200002070-00024, 10674483

[ref30] Isenbrandt A. Morissette M. Bourque M. Lamontagne-Proulx J. Coulombe K. Soulet D. . (2021). Effect of sex and gonadectomy on brain MPTP toxicity and response to dutasteride treatment in mice. Neuropharmacology 201:108784. doi: 10.1016/j.neuropharm.2021.108784, 34555366

[ref31] Jarras H. Bourque M. Poirier A. A. Morissette M. Coulombe K. Di Paolo T. . (2020). Neuroprotection and immunomodulation of progesterone in the gut of a mouse model of Parkinson's disease. J. Neuroendocrinol. 32:e12782. doi: 10.1111/jne.12782, 31430407

[ref32] Jia F. Fellner A. Kumar K. R. (2022). Monogenic Parkinson's disease: genotype, phenotype, pathophysiology, and genetic testing. Genes (Basel) 13:471. doi: 10.3390/genes13030471, 35328025 PMC8950888

[ref33] Kalia L. V. Lang A. E. (2015). Parkinson's disease. Lancet 386, 896–912. doi: 10.1016/S0140-6736(14)61393-3, 25904081

[ref34] Koebele S. V. Bimonte-Nelson H. A. (2016). Modeling menopause: the utility of rodents in translational behavioral endocrinology research. Maturitas 87, 5–17. doi: 10.1016/j.maturitas.2016.01.015, 27013283 PMC4829404

[ref35] Kulkarni J. Gavrilidis E. Worsley R. Van Rheenen T. Hayes E. (2013). The role of estrogen in the treatment of men with schizophrenia. Int. J. Endocrino.l Metab. 11, 129–136. doi: 10.5812/ijem.6615, 24348584 PMC3860106

[ref36] Lee W. L. Cheng M. H. Tarng D. C. Yang W. C. Lee F. K. Wang P. H. (2013). The benefits of estrogen or selective estrogen receptor modulator on kidney and its related disease-chronic kidney disease-mineral and bone disorder: osteoporosis. J. Chin. Med. Assoc. 76, 365–371. doi: 10.1016/j.jcma.2013.03.010, 23664736

[ref37] Litim N. Bourque M. Al Sweidi S. Morissette M. Di Paolo T. (2015). The 5alpha-reductase inhibitor Dutasteride but not finasteride protects dopamine neurons in the MPTP mouse model of Parkinson's disease. Neuropharmacology 97, 86–94. doi: 10.1016/j.neuropharm.2015.05.015, 26006269

[ref38] Litim N. Morissette M. Caruso D. Melcangi R. C. Di Paolo T. (2017a). Effect of the 5alpha-reductase enzyme inhibitor dutasteride in the brain of intact and parkinsonian mice. J. Steroid Biochem. Mol. Biol. 174, 242–256. doi: 10.1016/j.jsbmb.2017.09.021, 28982631

[ref39] Litim N. Morissette M. Di Paolo T. (2016). Neuroactive gonadal drugs for neuroprotection in male and female models of Parkinson's disease. Neurosci. Biobehav. Rev. 67, 79–88. doi: 10.1016/j.neubiorev.2015.09.024, 26708712

[ref40] Litim N. Morissette M. Di Paolo T. (2017b). Effects of progesterone administered after MPTP on dopaminergic neurons of male mice. Neuropharmacology 117, 209–218. doi: 10.1016/j.neuropharm.2017.02.007, 28192111

[ref41] Lu J. Xian T. J. Li C. J. Wang Y. (2025). The estrogen-brain interface in neuroinflammation: a multidimensional mechanistic insight. Front. Aging Neurosci. 17:1671552. doi: 10.3389/fnagi.2025.1671552, 40959795 PMC12434045

[ref42] Morissette M. Al Sweidi S. Callier S. Di Paolo T. (2008). Estrogen and SERM neuroprotection in animal models of Parkinson's disease. Mol. Cell. Endocrinol. 290, 60–69. doi: 10.1016/j.mce.2008.04.008, 18515001

[ref43] Olanow C. W. Stocchi F. (2018). Levodopa: a new look at an old friend. Mov. Disord. 33, 859–866. doi: 10.1002/mds.27216, 29178365

[ref44] Ou Z. Pan J. Tang S. Duan D. Yu D. Nong H. . (2021). Global trends in the incidence, prevalence, and years lived with disability of Parkinson's disease in 204 countries/territories from 1990 to 2019. Front. Public Health 9:776847. doi: 10.3389/fpubh.2021.776847, 34950630 PMC8688697

[ref45] Pesce G. Artaud F. Roze E. Degaey I. Portugal B. Nguyen T. T. H. . (2022). Reproductive characteristics, use of exogenous hormones and Parkinson disease in women from the E3N study. Brain 146, 2535–2546. doi: 10.1093/brain/awac440, 36415953 PMC10232244

[ref46] Poirier A. A. Cote M. Bourque M. Jarras H. Lamontagne-Proulx J. Morissette M. . (2022). Differential contribution of estrogen receptors to the intestinal therapeutic effects of 17beta-estradiol in a murine model of Parkinson's disease. Brain Res. Bull. 187, 85–97. doi: 10.1016/j.brainresbull.2022.06.019, 35781029

[ref47] Poirier A. A. Cote M. Bourque M. Morissette M. Di Paolo T. Soulet D. (2016). Neuroprotective and immunomodulatory effects of raloxifene in the myenteric plexus of a mouse model of Parkinson's disease. Neurobiol. Aging 48, 61–71. doi: 10.1016/j.neurobiolaging.2016.08.004, 27644075

[ref48] Riederer P. Wuketich S. (1976). Time course of nigrostriatal degeneration in Parkinson's disease. A detailed study of influential factors in human brain amine analysis. J. Neural Transm. 38, 277–301. doi: 10.1007/BF01249445 956814

[ref49] Rocca W. A. Bower J. H. Maraganore D. M. Ahlskog J. E. Grossardt B. R. de Andrade M. . (2008). Increased risk of parkinsonism in women who underwent oophorectomy before menopause. Neurology 70, 200–209. doi: 10.1212/01.wnl.0000280573.30975.6a, 17761549

[ref50] Rocca W. A. Smith C. Y. Gazzuola Rocca L. Savica R. Mielke M. M. (2022). Association of Premenopausal Bilateral Oophorectomy with Parkinsonism and Parkinson Disease. JAMA Netw. Open 5:e2238663. doi: 10.1001/jamanetworkopen.2022.38663, 36287560 PMC9606839

[ref51] Santoro N. Roeca C. Peters B. A. Neal-Perry G. (2021). The menopause transition: signs, symptoms, and management options. J. Clin. Endocrinol. Metab. 106, 1–15. doi: 10.1210/clinem/dgaa764, 33095879

[ref52] Trabert B. Sherman M. E. Kannan N. Stanczyk F. Z. (2020). Progesterone and breast Cancer. Endocr. Rev. 41, 320–344. doi: 10.1210/endrev/bnz001, 31512725 PMC7156851

[ref53] Veenman L. (2020). Raloxifene as treatment for various types of brain injuries and neurodegenerative diseases: a good start. Int. J. Mol. Sci. 21:7586. doi: 10.3390/ijms21207586, 33066585 PMC7589740

[ref54] Wang Q. Dong X. Wang Y. Li X. (2018). Raloxifene as an adjunctive treatment for postmenopausal women with schizophrenia: a meta-analysis of randomized controlled trials. Arch. Womens Ment. Health 21, 31–41. doi: 10.1007/s00737-017-0773-2, 28849318

[ref55] Willard A. M. Bouchard R. S. Gittis A. H. (2015). Differential degradation of motor deficits during gradual dopamine depletion with 6-hydroxydopamine in mice. Neuroscience 301, 254–267. doi: 10.1016/j.neuroscience.2015.05.068, 26067595 PMC4527082

[ref56] Wright D. W. Kellermann A. L. Hertzberg V. S. Clark P. L. Frankel M. Goldstein F. C. . (2007). ProTECT: a randomized clinical trial of progesterone for acute traumatic brain injury. Ann. Emerg. Med. 49:391-402, 402 e391-392. doi: 10.1016/j.annemergmed.2006.07.932, 17011666

[ref57] Xiao G. Wei J. Yan W. Wang W. Lu Z. (2008). Improved outcomes from the administration of progesterone for patients with acute severe traumatic brain injury: a randomized controlled trial. Crit. Care 12:R61. doi: 10.1186/cc6887, 18447940 PMC2447617

[ref58] Xu R. Wang X. Zhang J. Ren Y. Su L. Sang L. . (2026). Safety profile of progesterone: insights from an FDA adverse event reporting system (FAERS)-based pharmacovigilance study. J. Int. Med. Res. 54:3000605261417447. doi: 10.1177/03000605261417447, 41655271 PMC12883713

[ref59] Zarate S. M. Garcia R. C. Pandey G. Srinivasan R. (2025). Systemically circulating 17beta-estradiol enhances the neuroprotective effect of the smoking cessation drug cytisine in female parkinsonian mice. NPJ Parkinsons Dis. 11:6. doi: 10.1038/s41531-024-00855-3 PMC11698717 39753582

[ref60] Zhu X. M. Zheng W. Li X. H. Cai D. B. Yang X. H. Ungvari G. S. . (2018). Adjunctive raloxifene for postmenopausal women with schizophrenia: a meta-analysis of randomized, double-blind, placebo-controlled trials. Schizophr. Res. 197, 288–293. doi: 10.1016/j.schres.2018.01.017, 29395611

[ref61] Zsido R. G. Williams A. N. Barth C. Serio B. Kurth L. Mildner T. . (2023). Ultra-high-field 7T MRI reveals changes in human medial temporal lobe volume in female adults during menstrual cycle. Nat. Ment. Health 1, 761–771. doi: 10.1038/s44220-023-00125-w

