# Supplementary material for: Raloxifene and progesterone neuroprotection in a female mouse model of Parkinson’s disease
Source: Front Neurosci. 2026 Sep 9;20:1870069. doi: 10.3389/fnins.2026.1870069 (PMC13597706; doi:10.3389/fnins.2026.1870069)
Supplement: Supplementary file 1 [file Data_Sheet_1.PDF]

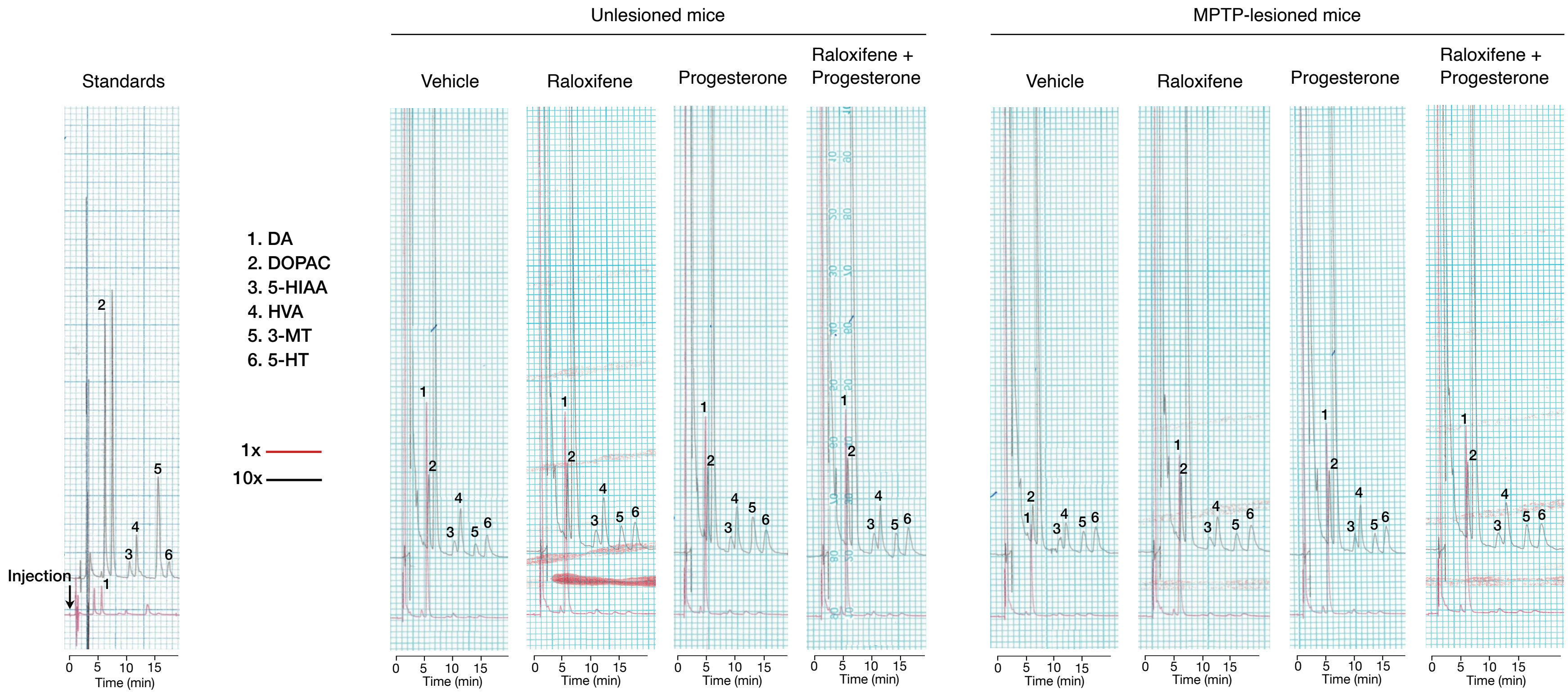

Supplementary Figure 1. Typical HPLC with electrochemical detection chromatograms of a standard mixture solution (at a concentration of 0.6 ng for each compound) and mouse striatum sample showing each treatment condition. Output signal was recorded with two different levels of magnification (1X and 10X). The electrochemical potential was set at 0.8 V with respect to an Ag/AgCl reference electrode (range 10 nA). The results in ng/mg of protein are shown in Figure 2.
